# Thermal Error Prediction in High-Power Grinding Motorized Spindles for Computer Numerical Control Machining Based on Data-Driven Methods

**DOI:** 10.3390/mi16050563

**Published:** 2025-05-07

**Authors:** Quanhui Wu, Yafeng Li, Zhengfu Lin, Baisong Pan, Dawei Gu, Hailin Luo

**Affiliations:** 1 College of Mechanical Engineering, Zhejiang University of Technology, Hangzhou 310023, China; wuqh@zjut.edu.cn (Q.W.); goodavid@zjut.edu.cn (D.G.); 221122020447@zjut.edu.cn (H.L.); 2 Key Laboratory of Special Purpose Equipment and Advanced Processing Technology, Ministry of Education and Zhejiang Province, Zhejiang University of Technology, Hangzhou 310023, China; 3 Zhejiang Zomax Transmission Co., Ltd., Wenling 317513, China; lzf730505@126.com

**Keywords:** motorized spindle, thermal error, ensemble learning, prediction model

## Abstract

The thermal error of the high-power grinding motorized spindle, caused by heating, seriously affects machining accuracy. In this paper, an ensemble learning algorithm is used to predict the thermal error of a high-precision motorized spindle. The subsequent problem of thermal error compensation can be effectively solved by a suitable thermal error model, which is crucial for improving the machining accuracy of the actual machining process. Firstly, the steady-state temperature field of the grinding motorized spindle is analyzed and used to determine the position of the sensors. Then, a signal acquisition instrument is used to monitor real-time temperature data. After that, experimental results are obtained, followed by verification. Finally, based on experimental data and the optimization results of temperature measurement points, temperature data are used as the input variable, and thermal deformation data are used as the output variable. The ensemble learning model is composed of different weak learners, which include multiple linear regression, back-propagation, and radial basis function neural networks. Different weak learners are trained using datasets separately, and the output of the weak learners is used as input to the model. Through integrating strategies, an ensemble learning model is established and compared with a weak learner. The error residual set of the ensemble learning model remains within [−0.2, 0.2], and the prediction performance shows that the ensemble learning model has a better predictive effect and strong robustness.

## 1. Introduction

Thermal deformation is the main factor that influences machining accuracy, and almost 40–70% of machining accuracy errors in Computer Numerical Control machine tools are caused by thermal errors [1,2]. The grinding motorized spindle, serving as the primary power source for Computer Numerical Control machine tools, produces thermal error that accounts for 50% of the total thermal errors, greatly reducing machining accuracy [3]. Compared with other methods for controlling thermal error, error compensation has the characteristics of good economy and high applicability, and revealing the thermal deformation mechanism is crucial for the thermal error compensation of the motorized spindle [4]. Thermal deformation effects change with variationsin cutting parameters, including spindle speed, machining parameters, and so on. Thus, it is essential to develop a thermal error prediction model for the motorized spindle to accurately predict the thermal error during the machining process [5].

With the widespread application of CNC machine tools, numerous scholars have carried out extensive research on the thermal deformation theory of motorized spindles [6,7]. Significantly, an accurate thermal error model of the motorized spindle is established to reveal the relationship between temperature and thermal deformation based on different mathematical methods and principles. Yin et al. [8] proposed a selective ensemble BP neural network-based thermal error technique for the motorized spindle. Li et al. [9] optimized the motorized spindle using the particle swarm optimization technique. Li et al. [10] created a model for predicting spindle heat errors using a modified particle swarm optimization BP neural network. Li et al. [11,12] established a least squares support vector machine prediction model optimized by the Aquila Optimizer and compared it with a particle swarm optimization model. Wu et al. [13] calculated axial and radial thermal errors using deep learning convolutional neural networks. Li et al. [14] established a model for predicting thermal errors using a Nonlinear Auto-Regressive model with Exogenous Inputs neural network. In summary, it has gradually evolved from the initial mechanistic model of the motorized spindle to a data-driven model, and artificial intelligence algorithms are increasingly being used in the thermal error model. Abdulshaod et al. [15] established a thermal error prediction model based on an adaptive neural fuzzy inference system to explore the relationship between temperature changes and thermal errors in milling machines. Experimental verification results showed that the thermal error prediction model established by this method had better prediction performance than artificial neural networks.

The thermal deformation of the motorized spindle plays a critical role in determining overall machining accuracy. The thermal error impacts can be effectively reduced through thermal error compensation of the motorized spindle. Zimmermann et al. [16] developed a new method for adaptive model input by combining the group least absolute shrinkage and selection operator with the particle swarm optimization algorithm-optimized autoregressive model. This method improves the self-optimization ability of the thermal error prediction model and enables accurate and robust long-term predictions of thermal errors. Li et al. [17] proposed a sparrow search algorithm to predict the thermal errors of the motorized spindle. Guo et al. [18] examined both the measurement and compensation of the spindle thermal error in two turntable machine tools and presented an experimental heat source measurement method. Bao et al. [19] introduced a predictive model approach for thermal errors in numerical control machines, leveraging a BP neural network and the beetle antennae search algorithm. Fu et al. [20] proposed an ensemble model of the motorized spindle that combines temperature-sensitive points. Fan et al. [21] introduced a novel method for predicting the thermal deformation of a grinding machine spindle by utilizing the principles of heat energy conduction in conjunction with neural network techniques. Cheng et al. [22] presented an effective model for predicting thermal errors using a convolutional neural network and long short-term memory.

The aforementioned thermal error prediction models have developed appropriate algorithms to address their respective issues; however, there has been limited consideration of establishing enhanced robust prediction models that are applicable across different operating conditions. Given that the thermal state of the motorized spindle is highly nonlinear and unstable, it is of significant importance to develop a thermal error prediction model with enhanced robustness. The BP neural network, proposed by Rumelhart et al. [23] in 1986, is a feed-forward neural network that is trained using the error back-propagation algorithm. It has been widely applied in the field of regression prediction. However, the BP neural network often faces issues of slow convergence and getting stuck in local optima during the solving process [24]. In addition, the traditional prediction model cannot achieve high efficiency when dealing with massive data. Compared with other algorithms, the proposed ensemble learning algorithm is an efficient and reliable method that prevents overfitting and leverages parallel and distributed computation to reduce running time [25,26]. In this study, a thorough analysis of the heat sources in the grinding motorized spindle is conducted, and the temperature field, as well as thermal deformation, are simulated using Ansys Workbench 2020. To address the issue of insufficient robustness in traditional single-model predictions, this paper proposes a thermal error prediction method based on ensemble learning. The method selects three simple models—multiple linear regression, BP neural network, and RBF neural network—as weak learners. These weak learners are trained using experimental data, and the output results from each are integrated through an ensemble learning model to obtain the final thermal error prediction. This approach significantly enhances the accuracy and robustness of predictions, providing a reliable theoretical foundation and technical support for subsequent research on thermal error compensation in grinding motorized spindles.

## 2. Mechanical Structure and Heat Source Calculation of the Motorized Spindle

### 2.1. Structural Characteristics of the Motorized Spindle

**Figure 1:**
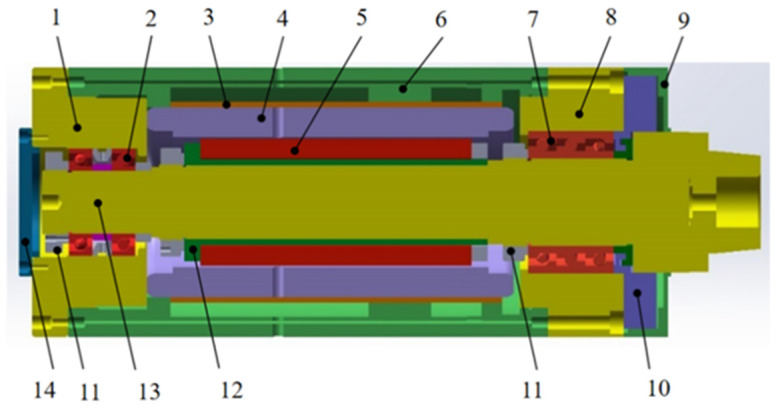
Schematic diagram of the internal mechanical structure of the motorized spindle. 1 Rear bearing seat, 2 rear bearings, 3 cooling sleeves, 4 motor stator, 5 motor rotor, 6 spindle housing, 7 front bearing, 8 front bearing seat, 9 rear end cover, 10 rear pressure cover, 11 isolation ring, 12 rotor inner sleeve, 13 core shaft, and 14 rear end cover.

The motorized spindle features a mechanical structure in which a motor drives the spindle shaft’s rotation directly. This structure includes a core shaft, motor rotor, motor stator, motor cooling sleeve, front and rear bearings, bearing seat, housing, and end cover. The basic mechanical structure of the motorized spindle is shown in Figure 1.

### 2.2. Analysis and Calculation of Heat Generation in the Motorized Spindle

#### 2.2.1. Analysis and Calculation of Motor Heat Generation

The heat generation of the motor is mainly related to power and efficiency, and the heat generation power of the motor can be calculated using Equation (1). The mechanical efficiency of the spindle motor is 90%, and the conversion efficiency of heat in the motor loss is 88%. Research shows that 1/3 of the heat loss of the motor is generated by the motor rotor, and 2/3 of the heat is generated by the motor stator. The heat generation rate of the motorized spindle motor can be calculated using Equation (2).

(1) $$Q=P_{N}(1-\eta )\times \eta^{\prime }$$

(2) $$q=\frac{Q}{V}$$

Here, *Q* is the heat generation power of the motor, KW; *η* is the mechanical efficiency of the spindle motor; *P_N_* is the rated power of the spindle motor, KW; *η*′ is the conversion efficiency of heat in motor losses; *q* is the heat generation rate of the motor, W/m^3^; and *V* is the volume of the heat source, m^3^.

#### 2.2.2. Analysis and Calculation of Bearing Heat Generation

As another part of the heat source for the motorized spindle, the heat generated by the bearing can be calculated using Equation (3). Due to the correlation between the friction torque of bearings and bearing load, speed, etc., as well as the possibility of unstable load and speed during operation, it is difficult to calculate an accurate fixed value. Usually, an approximate calculation method is used to roughly estimate the friction torque, *M*, of the bearings. The empirical formula is given in Equation (4). For angular contact bearings, the calculation method for *M_l_* is provided in Equation (5), where *Mv* is the friction torque related to the lubricant, which can be calculated using Equation (6).

(3) $$Q_{b}=\frac{2\pi }{60}nM$$

(4) $$M=M_{l}+M_{v}$$

(5) $$M_{l}=f_{1}P{\left(\frac{P}{C_{0}}\right)}^{c}d_{m}$$

(6) $$M_{v}=\left\{\begin{array}{c} 10^{-7}f_{0}(vn{)}^{2/3}d_{m}^{3},v\times n\geq 2000\\ 160*10^{-7}f_{0}d_{m}^{3},v\times n\leq 2000 \end{array}\right.$$

Here, *M* is the bearing friction torque, N·m; *n* is the spindle rotation speed, r/min; *Q_b_* is the heat generation power of the bearing, W; *M_l_* is the friction torque caused by external load, N·mm; *M_v_* is the friction torque related to the viscosity of the lubricant, N·m; *f*_0_ is the empirical coefficient related to the bearing lubrication method; *f*_1_ is the empirical coefficient related to bearings and loads; *P* is the equivalent load acting on the angular contact bearing, N; *C*_0_ is the basic rated static load of the bearing, N; *C* is related to the type of bearing, which can be obtained by consulting the bearing manual; *d_m_* is the bearing diameter, m; and *v* is the kinematic viscosity of the lubricant, m^2^/s.

### 2.3. Analysis and Calculation of Boundary Conditions in the Motorized Spindle

#### 2.3.1. Convective Heat Transfer Between the Stator and the Coolant

The heat transfer from the stator to the coolant of the motorized spindle involves forced convection heat transfer, and the heat transfer coefficient varies depending on the flow state of the cooling oil. After the coolant enters the cooling pipe, different flow rates show different flow patterns, and the cooling effects on the motorized spindle are also different. Therefore, the coolant flow pattern needs to be determined by the Reynolds coefficient, with the specific calculation provided in Equation (7), and the geometric characteristics calculated as shown in Equation (8). The Prandtl number depends on the fluid material parameters; different fluids have different Prandtl numbers, and the Prandtl number is calculated as indicated Equation (9).

(7) Re=uDv

(8) $$D=\frac{4A}{X}$$

(9) $$\mathrm{Pr}=\frac{c\cdot \mu }{\lambda }$$

Here, *u* is the characteristic velocity of the coolant, m/s; *D* is the scale of geometric characteristics, m; *v* is the kinematic viscosity of the coolant, m^2^/s; *A* is the cross-sectional area of the flow, m^2^; Pr is Prandtl number, J/(kg·°C); *μ* is the dynamic fluid viscosity, Pa·s; and *λ* is the thermal conductivity of the fluid, W/(m·k).

#### 2.3.2. Convective Heat Transfer Between the Spindle Housing and the Air

This part addresses natural convection heat transfer, specifically calculated using Equation (10).

(10) $$h_{5}=N_{u}*\lambda /d_{shell}$$

Here, *h*_5_ is the convective heat transfer coefficient between the spindle housing shell and the air, W/(m^2^·°C); *d_shell_* is the diameter of the shell, m; *λ* is the thermal conductivity of the air, W/(m·k); and *N*_u_ is the Nusselt heat transfer coefficient.

#### 2.3.3. Heat Transfer in the Air Gap Between the Motor Stator and Motor Rotor

The convective heat transfer between the motor rotor and stator in a laminar flow state is calculated using Equations (11)–(15).

(11) $$h_{6}={\left(\frac{Nu\lambda }{d_{m}}\right)}^{0.25}$$

(12) $$d_{m}=0.5(D_{1}+D_{2})$$

(13) Nu=0.23(2δD2)0.25⋅Re0.5

(14) Re=uδν

(15) $$u=\frac{\pi nd}{60}$$

Here, *h*_6_ is the convective heat transfer coefficient of the air gap between the rotor and stator in a turbulent state, W/(m^2^·°C); *d_m_* is the average diameter of the air gap, m; *λ* is the air thermal conductivity, 0.0267 W/(m·°C); *D*_1_ is the diameter of the stator inner circle, m; *D*_2_ is the outer diameter of the rotor, m; Re is the Reynolds number; *v* is the aerodynamic viscosity, 1.48 × 10^−5^ m^2^/s; *u* is the fluid velocity, m/s; and *δ* is the thickness of the air gap, m.

### 2.4. Simulation of the Thermal Characteristics of the Motorized Spindle

#### 2.4.1. Establishment of a Finite Element Model

**Figure 2:**
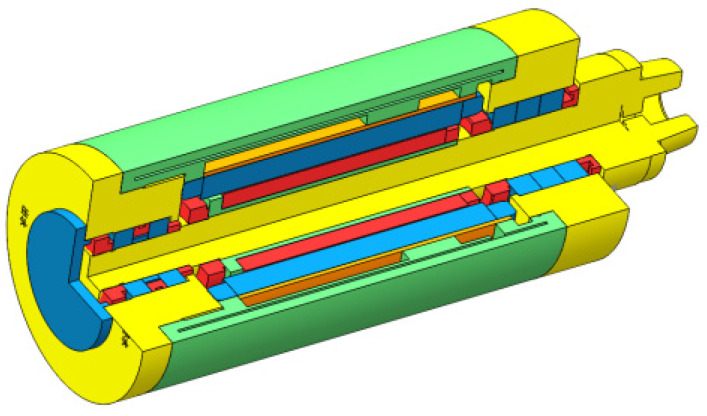
Simplified model of the motorized spindle.

**Figure 3:**
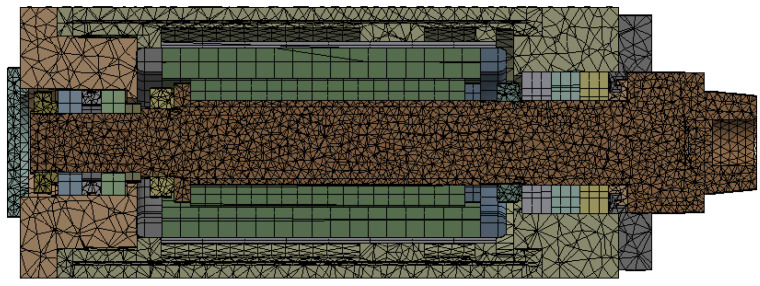
Mesh elements of the motorized spindle’s sectional view.

The finite element method is used to study the temperature changes of the motorized spindle during operation, exploring the changes in its temperature field and thermal deformation. The impact of temperature changes on the motorized spindle system is analyzed using ANSYS Workbench 2020, which provides steady-state and transient thermal analysis modules. Small structures such as threaded holes, air holes, oil holes, chamfers, fillets, and undercuts that are present in the model can be directly simplified. The motor stator and rotor of the motorized spindle can be simplified into cylinders of the same thickness. The simplified model of the motorized spindle is shown in Figure 2. The material parameters for each component of the motorized spindle include material density, elastic modulus, Poisson’s ratio, thermal conductivity, coefficient of thermal expansion, and specific heat capacity. The specific material parameters are shown in Table 1. The motorized spindle model is divided into a total of 97,739 elements, with 180,634 nodes. Usually, skewness should not be greater than 0.5, the Jacobian ratio should not be greater than 10, and the grid division should meet the usage requirements. The grid division results for the motorized spindle’s sectional view are shown in Figure 3.

The thermal load and boundary conditions for the motorized spindle model should be established. According to the analysis results of the spindle shaft heat source and internal heat transfer, it is necessary to apply heating loads to the motor stator, motor rotor, and front and rear bearings and to set corresponding convection coefficients for the existing thermal convection. When the speed of the motor spindle is 3000 r/min, the heat generation rates of the front bearings, rear bearings, stator, and rotor are calculated, and the specific heat generation coefficients are shown in Table 2. The convective coefficient is shown in Table 3. The corresponding heat generation coefficient and heat dissipation coefficient are assigned according to the parameters in the table. The number and time interval of sub-steps for transient analysis are set to solve the temperature field. The solution result of the transient temperature field is used as the external load for the transient structural field, which is then incorporated into the transient structural analysis, and corresponding constraint conditions are adopted to determine the thermal deformation result.

#### 2.4.2. Steady-State Thermal Analysis of the Motorized Spindle

**Figure 4:**
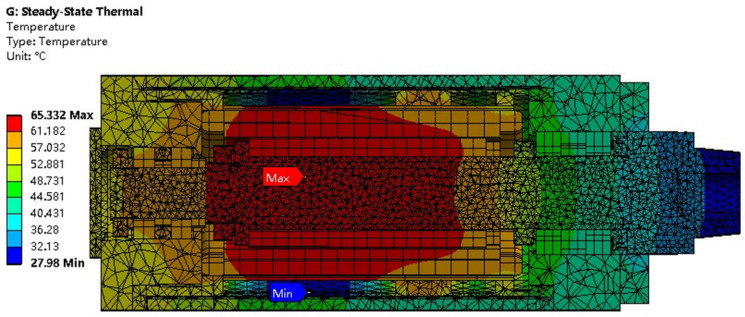
Steady-state temperature distribution of the grinding motorized spindle model.

The steady-state thermal analysis of the motorized spindle refers to the thermal analysis results obtained when the system reaches thermal equilibrium, at which point the temperature field no longer changes with time. Using the finite element analysis software Ansys Workbench 2020, the previously obtained thermal boundary parameters were applied to solve for the steady-state temperature field. The results of this steady-state thermal analysis are presented in Figure 4. Key temperature field information is clarified based on the results of the simulation analysis, providing a practical reference value for temperature data acquisition in conducting the experiment.

Through simulation results, it is observable that the spindle core attains the highest temperature of about 65.3 °C, whereas the cooling sleeve exhibits the lowest temperature point. The temperature rise of the spindle shaft is caused by its close proximity to the rotor and poor heat dissipation conditions, while the cooling sleeve carries a large amount of heat away by convection with the coolant, resulting in the lowest temperature of about 27.9 °C at the cooling sleeve. The overall temperature of the grinding motorized spindle shows a gradient decreasing trend extending outward from the spindle, and the spindle temperature generally meets the central symmetry along the spindle centerline, which is consistent with the central symmetry structure of the grinding motorized spindle. It is evident that the temperature variations are significant along the axial direction and comparatively minor along the circumferential direction. Consequently, in conducting experiments, the placement of temperature measuring points is primarily focused along the axial axis to ensure adequate collection of temperature data.

#### 2.4.3. Transient Thermal Analysis of the Motorized Spindle

**Figure 5:**
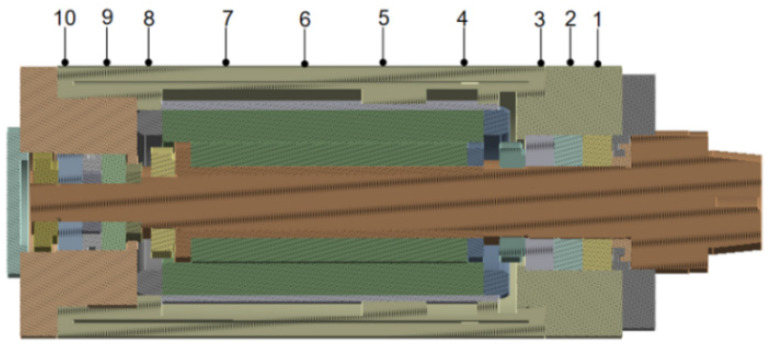
Schematic diagram of transient temperature measuring points.

**Figure 6:**
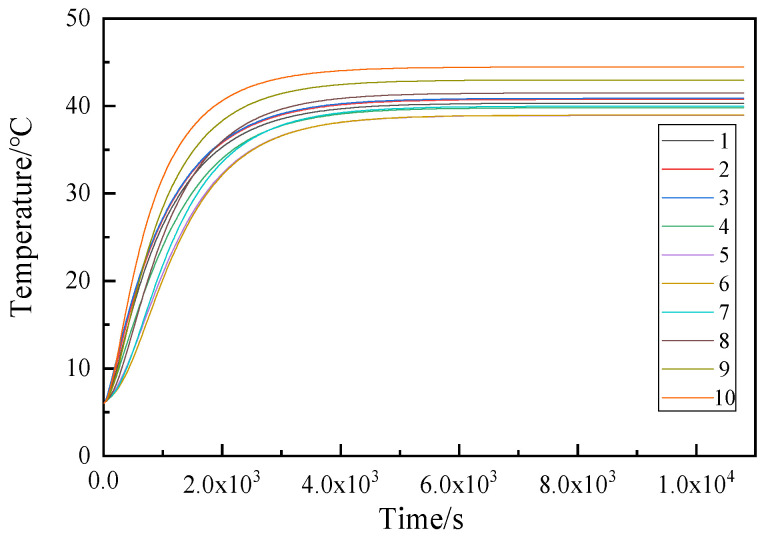
Curve of transient temperature.

The transient thermal analysis of the motorized spindle investigates the evolution of the temperature field as the system changes over time. To solve the transient temperature field, a transient thermal analysis simulation model for the motorized spindle is established. Ten temperature nodes on the housing surface of the motorized spindle are selected as the solution objects, with specific locations shown in Figure 5. The simulation duration for temperature was set to 11,000 s, and after solving the transient temperature field, the temperature variation curves at each temperature node were obtained, as shown in Figure 6. There are three front bearings, two rear bearings, and five stator–rotor nodes, which are uniformly distributed at their corresponding positions. From the transient temperature curves of the temperature nodes, it can be seen that the temperature curves of nodes at different positions on the motorized spindle shell show a phenomenon of initially rising and then tending to stabilize. The rate of temperature rise varies slightly between different temperature measurement points, and the time to reach a plateau and the final temperature are also slightly different, but the overall trend is consistent.

#### 2.4.4. Thermal Deformation Analysis of the Motorized Spindle

**Figure 7:**
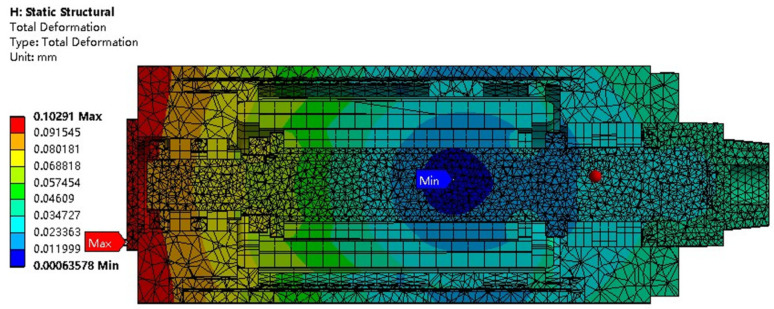
Steady-state thermal deformation field.

The thermal deformation of the motorized spindle is determined by utilizing the temperature field obtained from thermal analysis to compute the structural expansion and deformation induced by temperature variations. The temperature field simulation results are applied to the structural module as the load of the structural field, and corresponding constraints are set to solve the structural field of the motorized spindle. The overall deformation simulation results are shown in Figure 7. From the thermal deformation simulation results of the motorized spindle described above, it can be seen that the position with the largest deformation of the motorized spindle is at the rear end, and the position with the smallest deformation is near the front bearing. Overall, the deformation of the motorized spindle shows a clear stepped shape, starting from the position of the front bearing of the motorized spindle near the motor stator and motor rotor. As the distance from the left to right ends gradually increases, the thermal deformation also increases. The main reason for this phenomenon is that the front bearing of the motorized spindle adopts a positioning installation, while the rear bearing adopts a non-positioning installation. This installation method constrains the front and rear ends of the front bearing, while one end of the rear bearing is mutually constrained with the core shaft, and the other end is unrestrained, creating a gap. When thermal deformation occurs, the deformation extends to the less stressed end, which is the non-constrained end. As the distance increases, the deformation accumulates, resulting in the smallest deformation near the constrained end of the front bearing and larger deformation at the farther position.

## 3. Experiments of Thermal Error

### 3.1. Arrangement of Temperature and Displacement Sensors

In this experiment, temperature measurements were conducted using a K-type thermocouple, while thermal deformation was quantified using a Keyence LK-H025 laser displacement sensor, manufactured in Osaka, Japan. Data acquisition was performed using the DH5922D signal acquisition instrument, produced by Jiangsu Donghua Testing Technology Co., Ltd. (Taizhou, China).

**Figure 8:**
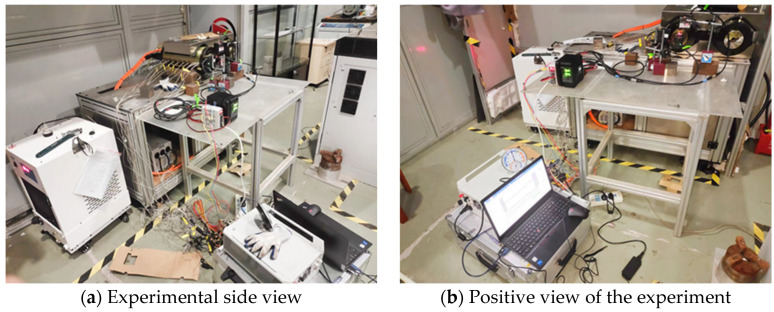
Motorized spindle experimental monitoring.

**Figure 9:**
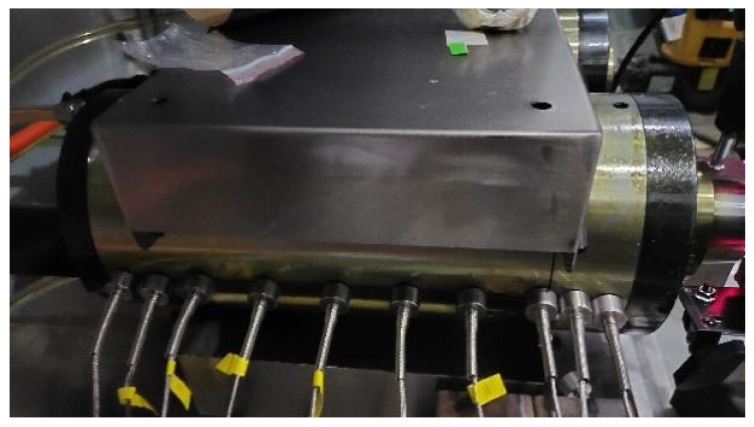
Layout of the temperature sensors.

**Figure 10:**
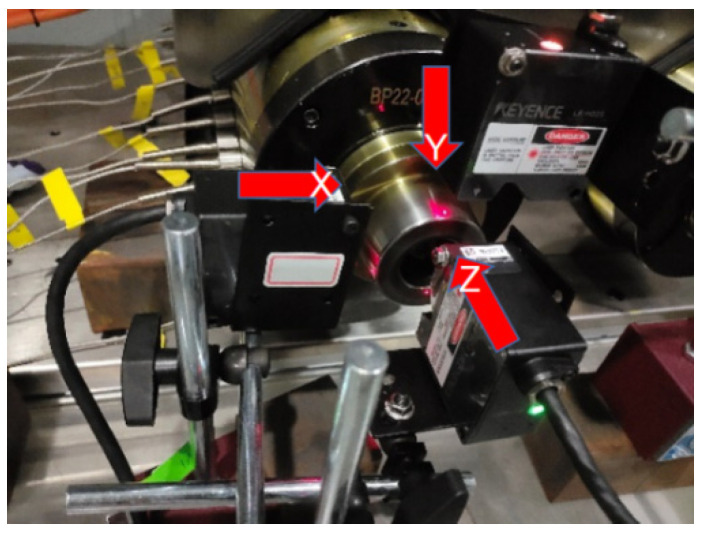
Layout of the displacement sensors.

The experimental setup and measurement circuit are shown in Figure 8. In the experiment, the location of the temperature measurement point of the motorized spindle corresponds to Figure 6, while the layout of the temperature sensors is shown in Figure 9. The temperature sensors are arranged in the front bearing area, stator rotor area, and rear bearing area. To measure the thermal deformation of the motorized spindle, a three-point approach is selected, as this study solely focuses on thermal displacement along three axis directions. Figure 10 shows the laser displacement sensor installation. The installed sensor is connected to the acquisition channel of the signal acquisition instrument, which, in turn, is connected to the upper computer. Subsequently, the acquisition instrument is activated in preparation for the experiment.

### 3.2. Experimental Data Collection and Analysis

In order to obtain the temperature and thermal deformation values of the motorized spindle under no-load conditions, this experiment is divided into three groups. For the no-load tests, the motorized spindle is configured to rotate at different speeds, and the experimental data are shown in Table 4.

**Figure 11:**
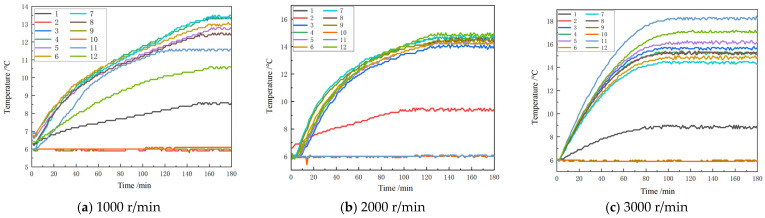
Curves of time and temperature at various rotational speeds.

For this experiment, 10 Hz is the frequency at which data are collected. As temperature and thermal deformation are slow variables, the measurement frequency does not need to be too high. As a result, data collection requires taking points at regular intervals. Taking into account the duration of the experiment and the amount of data, a data interval of 1 min is selected, so the data sampling interval is 600. After the collected temperature and thermal deformation data are output at 600 intervals, it is necessary to deal with sudden changes, overall translation, and other abnormalities in the data. After removing the initial point mutation and the overall translation of the data, the data are denoised to obtain the noise-removed temperature and thermal deformation data. Origin is then used to draw the corresponding time–temperature curve and time–thermal deformation curve. The curves of time and temperature at various rotational speeds are shown in Figure 11.

In the experiment, there are a total of 12 temperature measurement points, with temperature measurement point 1 specifically designated for measuring the coolant temperature, point 2 for the ambient temperature, and points 3 through 12 of the temperature measurement corresponding to points 1 through 10 in Figure 5. From the time–temperature curve shown above, it can be observed that the temperatures of three temperature measurement points in this experiment are essentially fixed values, namely, No. 2, No. 9, and No. 10. Among these, No. 2 is the ambient temperature, and No. 9 and No. 10 are the motor stator and rotor temperatures, respectively. Based on the curve of temperature versus time in the transient thermal results of Figure 6, it can be determined that there is no fixed value situation, leading to the conclusion that the data collected by these two temperature sensors are abnormal. Due to the fact that abnormal data cannot accurately reflect the temperatures of the main shaft stator and rotor, they are discarded. From the time–temperature curves at the three speeds mentioned above, the overall trend is a gradual increase that eventually stabilizes. The temperature rises quickly in the beginning, gradually climbs over time, and eventually stabilizes around a particular value. The difference in the time–temperature curve at different speeds lies in the rate of temperature rise, the time to reach the plateau, and the temperature value after reaching the plateau. The higher the spindle speed, the higher the heating rate, the greater the heat generated per unit time, the faster the temperature rises, and the shorter the time required for the temperature to reach a stable state in the early stage. Conversely, at relatively lower speeds, the temperature rises slowly and takes a long time to reach a stable state. In addition, it can be observed that the temperature trends are consistent by comparing the temperature variation results in Figure 11c with those in Figure 6. However, under experimental conditions, the temperature stabilizes at a lower value compared to the simulation results. This discrepancy is primarily due to the continuous variation of the internal coolant temperature in the motorized spindle, which cannot be accurately represented in the simulation by applying fixed boundary conditions.

**Figure 12:**
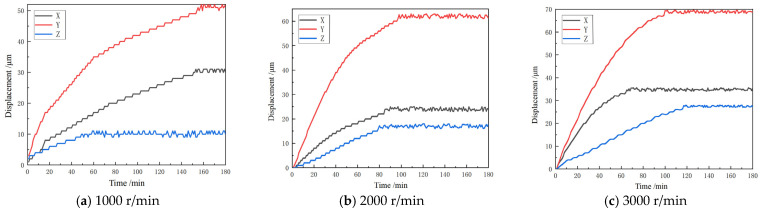
Time–thermal deformation curves at different speeds.

Thermal deformation data collected in the experiment are processed in the same way. After removing factors such as sudden changes and overall translation of the thermal deformation data, a reasonable curve fitting is performed on the thermal deformation data. Plots are generated to depict the time–thermal deformation curves of the spindle at various speeds using the data obtained from the curve fitting method, as shown in Figure 12.

The trend of the thermal deformation time–temperature curve obtained at various speeds is essentially the same, as shown in Figure 12. The thermal deformation in all three directions initially increases and then gradually stabilizes over time. At the same speed, the thermal deformation of the spindle shaft in the Y direction is the highest, followed by the X direction and the Z direction. The Y direction is the axial direction of the main axis, and according to the thermoelastic theorem, its maximum thermal deformation along the axis should be the largest. The X and Z directions are the radial directions of the main axis, and due to their small size, the thermal deformation is less than that in the Y direction. The spindle speed affects both the amount and rate of deformation, as well as the time taken to reach a stable condition. This is seen in the differences in the time–thermal deformation curves at different rotational speeds.

## 4. Modeling and Verification of the Thermal Error Based on Ensemble Learning

To achieve effective compensation for thermal errors in CNC machine tool spindles, it is essential to develop a thermal error prediction model with both high accuracy and strong robustness. However, existing models often struggle to satisfy these two requirements simultaneously. To address this challenge, this section proposes the adoption of an ensemble learning approach, which integrates multiple base models to enhance overall prediction accuracy and generalization capability. As a generalized modeling strategy, ensemble learning effectively compensates for the limitations of individual models in terms of accuracy and robustness.

### 4.1. Principle of the Ensemble Learning Thermal Characteristic Prediction Model

#### 4.1.1. Foundational Concepts

(1) Multiple linear regression

Multiple linear regression is a statistical technique used to model the relationship between a dependent variable and multiple independent variables [27]. The core principle involves predicting the value of the dependent variable through a linear combination of the independent variables. The core of establishing the multiple linear regression model is to construct the linear regression relationship between the dependent variable and the independent variable, and the general form of its mathematical model is as follows:

(16) $$y=\beta_{0}+\beta_{1}x_{1}+\beta_{2}x_{2}+\ldots +\beta_{p}x_{p}+\varepsilon$$

where y is the dependent variable; $x_{i}\;(i=1,2,\cdots ,p)$ is the independent variable; $\beta_{i}\;(i=0,1,\cdots ,p)$ is the regression coefficient of the independent variable; and ε is the error term.

(2) BP neural network

**Figure 13:**
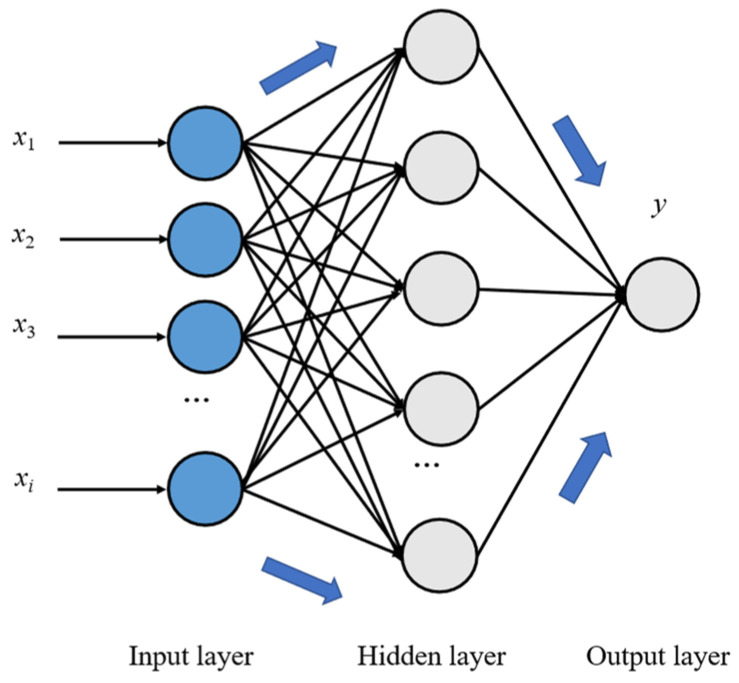
Structure of the BP neural network.

The architecture of the BP neural network [8], comprising an input layer, a hidden layer, and an output layer, is illustrated in Figure 13. The input layer is responsible for receiving data inputs, while the hidden layer serves as the core of the BP neural network, where complex computations and data processing occur through neuron connections. Finally, the output layer processes the data from the hidden layer and generates the predicted values.

In predicting the thermal error of the motorized spindle, the BP neural network uses the temperature and rotational speed of the spindle as input features. The network then extracts deep features from the data through the nonlinear activation function in the hidden layer. Finally, it outputs the predicted value of the thermal error. During the training process, the network iteratively adjusts the weights and biases using the backpropagation algorithm, minimizing the difference between the predicted values and the actual measurement errors. This process enables the accurate modeling and prediction of the thermal error in the motorized spindle.

(3) RBF neural network

Structurally, RBF neural networks are similar to BP neural networks, as both consist of an input layer, a hidden layer, and an output layer. The input layer directly transmits external inputs to the hidden layer, where each neuron corresponds to a radial basis function (typically a Gaussian function) that serves as a local response to the input space.

In predicting the thermal error of a motorized spindle, the RBF neural network can utilize its strong nonlinear approximation ability to accurately capture the complex mapping relationship between temperature change and thermal error, so as to realize the efficient and real-time prediction of the thermal error [28]. The steps for predicting thermal errors are as follows:

Step 1: Determine the network structure. Determine the number of grid nodes and the type of node basis functions.

Step 2: Determine the parameters. Determine the input and output vectors; initialize the connection weights, as well as the center and width vectors of the hidden layer neurons.

Step 3: Calculate the output value of the *i*th neuron in the hidden layer.

Step 4: Calculate the output of the output neuron.

Step 5: Iteratively calculatie the weight parameters.

#### 4.1.2. Principle of an Ensemble Learning Model

Due to the superior performance of the Stacking method compared to Bagging and Boosting, the Stacking ensemble algorithm is chosen for ensemble learning algorithm modeling. According to the steps of the Stacking modeling method, MATLAB 2020 is used to complete the integrated model training.

Before modeling the thermal error prediction of the motorized spindle, it is crucial to ensure the quality and reasonableness of the input data. Due to the inevitable presence of measurement errors, noise interference, and fluctuations in the acquisition conditions during the experimental process, the raw data often contain missing values and outliers. In order to improve the accuracy and stability of the subsequent model training, it is necessary to systematically preprocess the collected raw data. The specific preprocessing steps are described as follows:

Step 1: Process missing values and outliers. Check for missing values and outliers in the data, and eliminate samples that contain them.

Step 2: Data normalization. In order to avoid training bias caused by different feature scales, all input features and output thermal errors are normalized using min–max normalization, which linearly scales the data to the [0,1] interval to ensure that different features are of equal importance for model training.

Step 3: Dataset division. The training dataset uses optimized experimental data, taking the thermally sensitive X direction at different rotational speeds as an example. The input dataset for the model comprises temperature data and spindle speed from four temperature measurement points, 2, 3, 6, and 12, and the output dataset consists of thermal deformation data. The dataset is randomly divided into a training set and a test set in a ratio of 80% to 20%. The training set is used to train the model and optimize the parameters, and the test set is used to evaluate the generalization ability and prediction performance of the final model.

Step 4: Sub-model training data preparation. The training set is fed into three sub-models, including multiple linear regression, BP neural networks, and RBF neural networks, for independent training to obtain three different predictions. These predicted outputs will be used as first-level input features for the Stacking integration algorithm.

**Figure 14:**
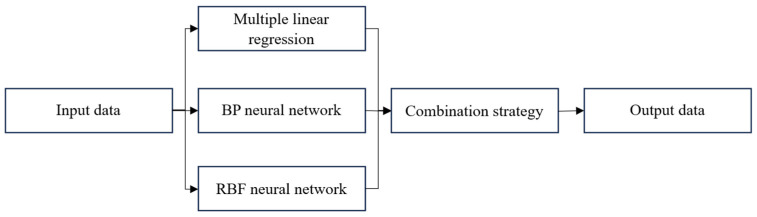
Basic model of ensemble learning.

The combination strategy employed in this paper integrates three sub-models: multiple linear regression, BP neural networks, and RBF neural networks. Each sub-model is independently trained on the input features and generates its own predictions. These predictions are then fed into a Stacking algorithm, where a meta-model is trained to combine them and produce the final prediction results. The basic principle of ensemble learning is shown in Figure 14.

The training dataset uses optimized experimental data, taking the thermally sensitive X direction at different rotational speeds as an example. The input dataset for the model comprises temperature data. The radial basis function model, BP neural network model, and multiple linear regression model are all trained using the abovementioned datasets. The predictive values are combined into a three-dimensional column vector, which serves as the input dataset for the meta model, which is divided into a training set and a testing set. Through the Integration Stacking strategy, the final response value of the integrated learning model is obtained.

### 4.2. Thermal Error Prediction and Validation of Ensemble Learning Models

#### 4.2.1. Thermal Error Prediction of an Ensemble Learning Model

Stacking is realized as follows: first, the data are k-fold sliced to obtain a *k*-fold dataset, in which *k* − 1 folds of the k-folds are used as the training set and the remaining one fold is used as the validation set. After the weak learner is trained on the training set, the validation set is used to validate the model, so as to obtain the one-fold prediction. The complete prediction is obtained by cycling through this process *k* times. This method is called the *k*-fold cross-validation method, which improves the generalization ability of the model. The predictions from different weak learners are then combined as input data for the next layer.

**Figure 15:**
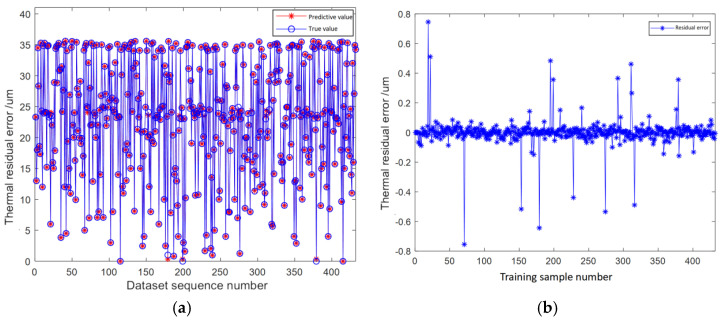
(**a**) Comparison chart of prediction results of the training set. (**b**) Residual graph of the training set.

The prediction results of the model for the training set are shown in Figure 15a. The training set makes up 80% of the dataset, with a total dataset of 432 data points. The prediction results graph of the training set reveals that there are no notable prediction points deviating significantly from the actual values, as the majority of predicted values closely align with the actual ones. The prediction accuracy of a single data point and the overall prediction accuracy of the training set can be determined by computing the residual value, which more intuitively reflects prediction accuracy. The residual graph of the ensemble learning model training set is shown in Figure 15b. From the graph, the overall residual values are within the range of [−0.8, 0.8], and the absolute residual values of the vast majority of data points are within the range of [0, 0.2] μm. Only a small number of data points have absolute residual values within [0.2, 0.8] μm. The overall prediction deviation of the training set is less than 1 um, indicating that the model training has achieved a satisfactory effect with a high degree of accuracy.

#### 4.2.2. Model Accuracy Verification

As the model is trained using experimental data, it is necessary to evaluate the training model quality. A common evaluation method is to test its accuracy, which can be verified through a validation set, and the evaluation of model accuracy is based on residuals. The validation set is input into the model as input data, and the model generates predicted data as the output. The residual error is obtained by subtracting the predicted data from the actual data, and the size of the residual error characterizes the model’s prediction accuracy.

**Figure 16:**
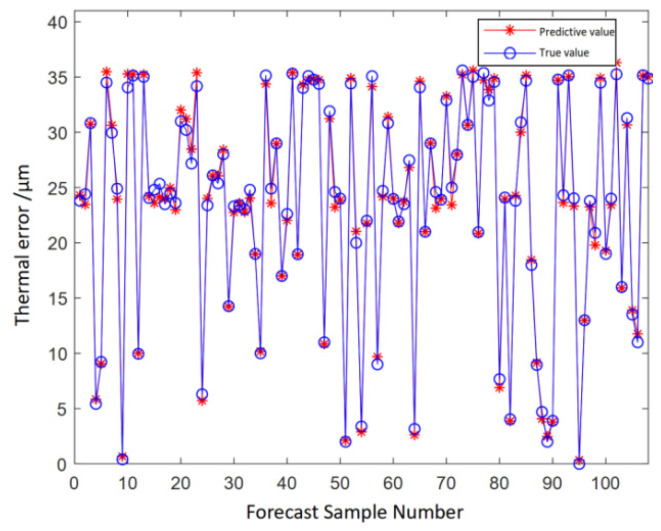
Comparison chart of the predicted results of the test set.

**Figure 17:**
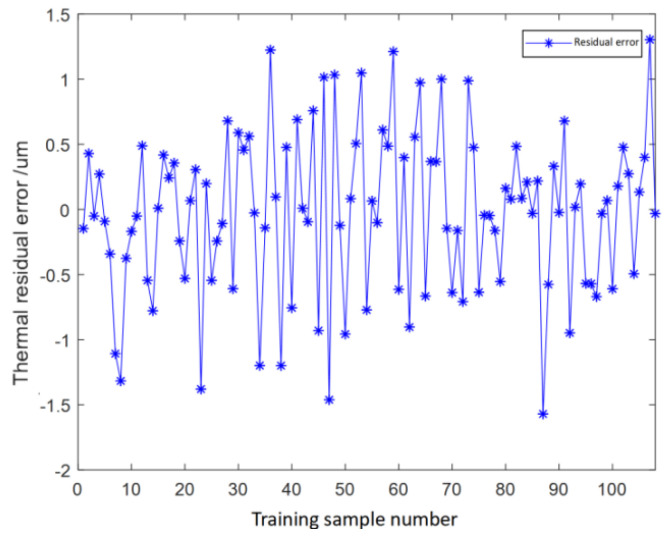
Residual plot of the test set.

The thermal error prediction model of the motorized spindle constructed based on the ensemble learning method outputs a final prediction value that synthesizes the results of each base learner. Figure 16 and Figure 17 show the predicted results and residual plots of the test set, respectively. After training the integrated model with the dataset mentioned above, the trained model is obtained. The training effect of the model appears to be good based on the prediction results of the training set. The trained model necessitates the utilization of a test set to validate its prediction accuracy. It is evident that the test set’s residual range falls within the interval of [−1.5, 1.6], the overall error is less than 2, and the results indicate that the prediction performance of the model is satisfactory.

**Figure 18:**
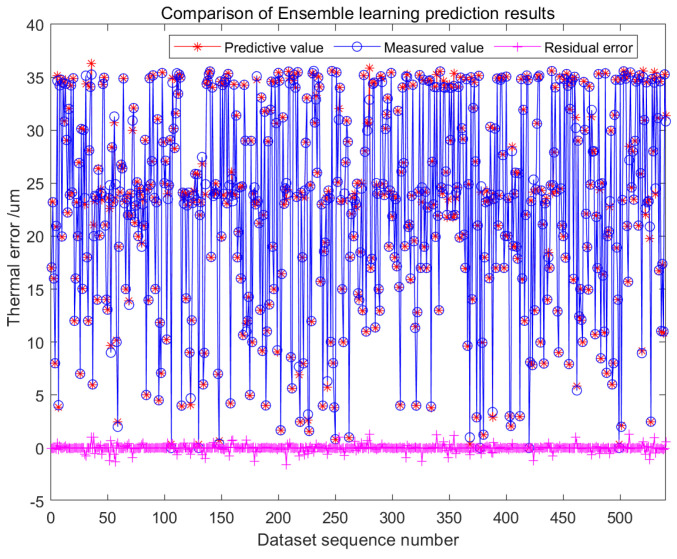
Comparison chart of the prediction results for ensemble learning.

**Figure 19:**
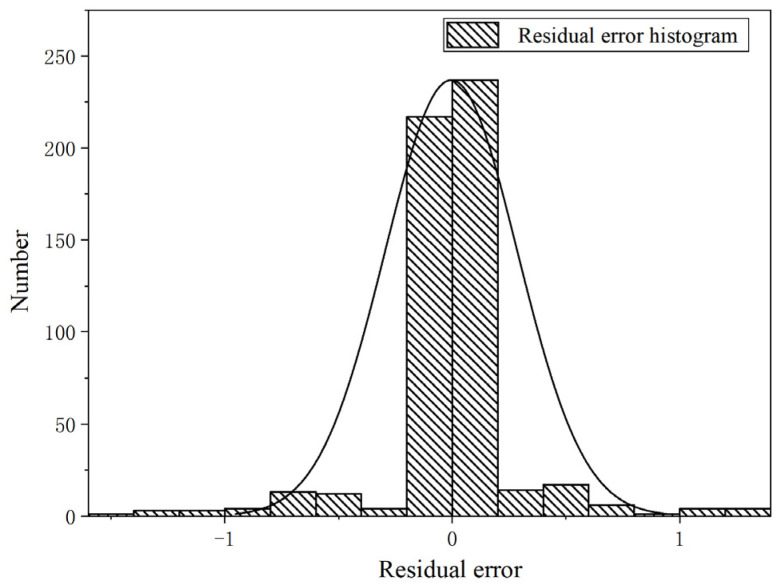
Residual error histogram for ensemble learning.

Figure 18 visually presents the overall prediction accuracy of the model. Figure 19 demonstrates that the residuals of the dataset follow a normal distribution, with values ranging between [−1.4, 1.6]. Notably, the majority of residuals fall within the narrower range of [−0.2, 0.2], indicating a high level of prediction accuracy.

#### 4.2.3. Testing Model Robustness

To determine the quality of the ensemble learning model, it is vital to first validate its accuracy and subsequently assess its robustness. This comprehensive verification process is essential for determining that the model has strong robustness. The robustness of a model refers to its anti-interference ability. To verify the model’s robustness, interference can be added to the input variables. When interference occurs in the input variables, high prediction accuracy indicates strong model robustness, whereas low prediction accuracy suggests weak robustness. To verify the robustness of the model, interference will be added to different input variables, and the prediction accuracy of the model will be observed when there is interference in a single variable and when all input variables have interference. If the model demonstrates high prediction accuracy, it indicates that the model has high robustness; if not, it indicates that the model has weak robustness.

**Figure 20:**
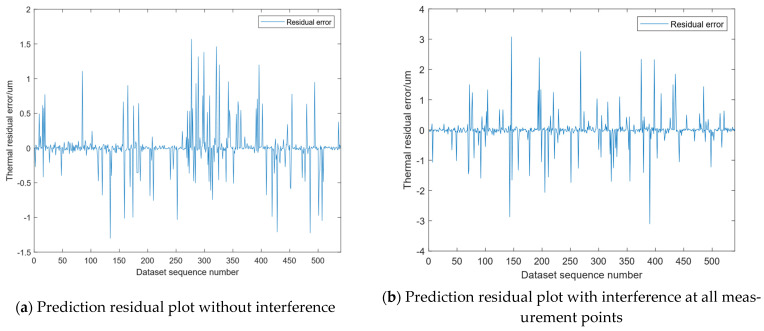

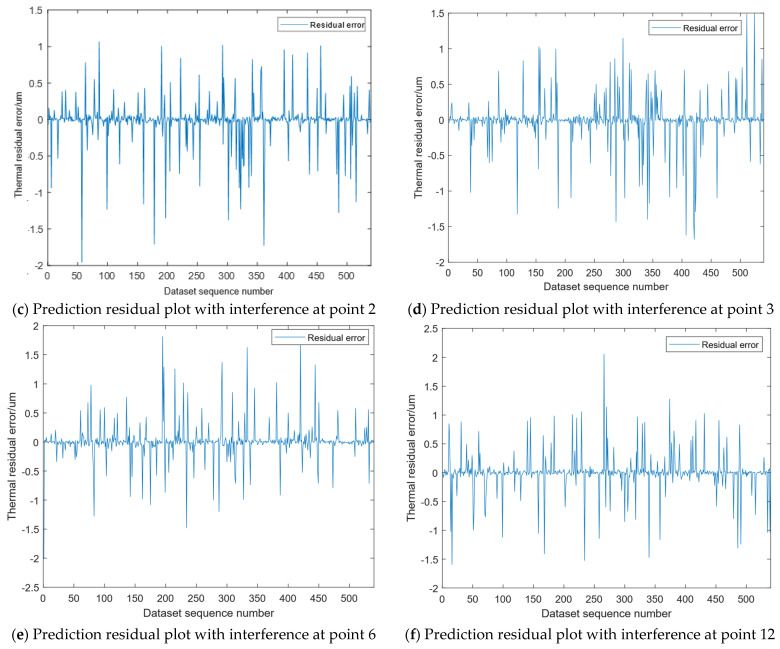
Comparison charts of prediction residuals for datasets under different interference conditions.

To demonstrate the superiority of model robustness, a thermal error residual graph is established based on the dataset, where the horizontal axis represents the sequence number of the predicted dataset and the vertical axis represents the corresponding thermal error residual for each dataset. The smaller the value corresponding to the vertical axis, the better the prediction accuracy of the model and the higher the robustness of the model. Figure 20 consists of six residual plots, including the residual plots without interference, the residual plots with interference from a single variable, and the residual plots with interference from all variables simultaneously. From the residual plot, the residual range of the dataset is [−1.5, 2] when there is no interference. The residual range is generally the same when a single variable has interference as when there is no interference. When all temperature variables have interference, the residual range is [−3, 3]. In order to compare the differences between the predicted values and the actual observed values, this study selected four performance indicators to quantify the accuracy and stability of the model. Table 5 shows the evaluation index of the model under different interference conditions.

According to Table 5, the model has the highest prediction accuracy. Although the prediction accuracy is slightly affected when there is interference, it is still relatively high. When all temperature variables have interference, the prediction accuracy is slightly lower than when only a single variable has interference. However, even when all measurement points are subject to interference, the model maintains a high prediction accuracy, demonstrating its strong robustness.

### 4.3. Comparative Modeling Methods

The ensemble learning model utilizes multiple basic models with average performance to complete the same task and summarizes the prediction results of these sub-models to generate an ensemble model that outperforms the basic models.

**Figure 21:**
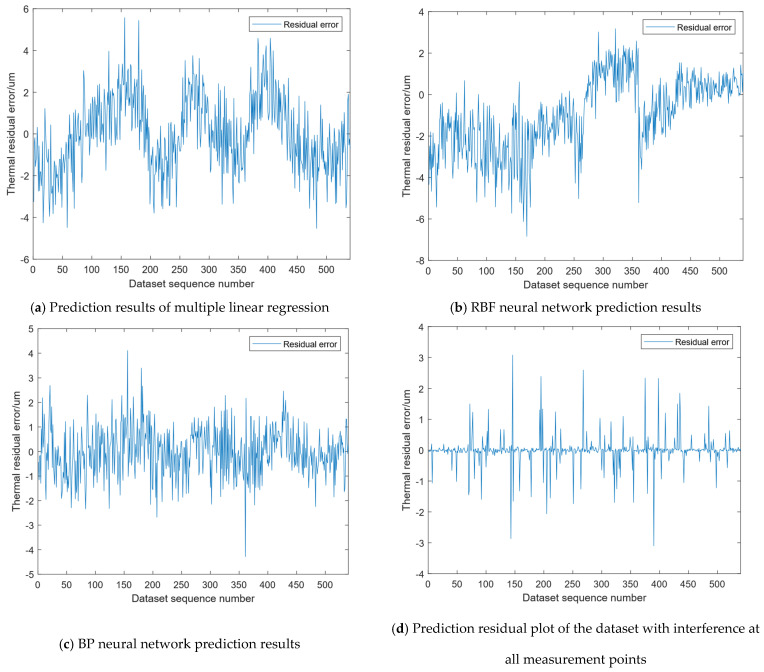
Comparison charts of prediction results of different models with interference.

Figure 21 shows the prediction residuals of different models in the presence of interference, including a comparison of the three types of residuals from the weak learner prediction results and the ensemble learning prediction results. According to the figure, the residual range of ensemble learning is [−3, 3], with the majority concentrated in [−1.5, 1.5]. The residual ranges for multiple linear regression, the RBF neural network, and the BP neural network are [−5, 6], [−7, 3], and [−4, 4], respectively. The residual error of multiple linear regression fluctuates periodically around 0, the RBF neural network residual is mainly distributed in [−5, 3], and the BP neural network residual is mainly distributed in [−2.5, 2]. From the perspective of residual distribution, ensemble learning exhibits superior prediction accuracy and robustness compared to its individual, weaker learners.

In order to gain an intuitive grasp of the performance disparities among various models, performance indicators can be used to quantify them. The performance parameters of the ensemble learning model and various weak learners in the presence of interference are calculated, as shown in Table 6.

In comparison to the three weak learners, the integrated learning model emerges as the superior choice. This finding indicates that the integrated learning model surpasses the three weak learners in terms of overall performance based on the characteristic indicators in the table.

## 5. Conclusions

This paper primarily focuses on the thermal error prediction of high-power grinding motorized spindles. To address the issues of low modeling accuracy and robustness in current methods, this study conducts an analysis of the heat generation and heat transfer from both internal and external heat sources of the grinding motorized spindle. Additionally, a simulation analysis of the temperature field and deformation field of the spindle’s thermal characteristics is performed. Based on the simulation results, temperature-sensitive points are identified, and experimental data is collected to construct a thermal error prediction model using an ensemble learning algorithm. The advantages of multiple algorithms are leveraged to resolve existing shortcomings. The conclusions are as follows:

(1) Three different types of commonly used regression algorithms are selected as weak learners for ensemble learning algorithms, and their modeling principles and implementation steps are introduced. The three models are trained using datasets, and the predicted values from the three trained models are combined and input into the metamodel as input datasets. The Stacking ensemble learning algorithm is used to integrate different weak learners to form a new ensemble learning model.
(2) The training of the ensemble learning model is complete, and the accuracy of the model is verified through a validation set. The results show that the model has high accuracy, the strong robustness of ensemble learning, and better prediction accuracy than weak learners. The proposed ensemble learning model can significantly improve predictive performance.

Experimental research on a single grinding motorized spindle has been conducted in this paper, and corresponding numerical control systems will be equipped according to its specifications to achieve subsequent applications in thermal error compensation. In addition, there are several factors that influence thermal error, such as material properties, load conditions, varying ambient temperature and humidity, etc. These factors will be further explored in future research.

## Figures and Tables

**Table 1:** Material parameters of the motorized spindle.

Corresponding Parts	Material	Elastic Modulus (GPa)	Density (kg/m^3^)	Specific Heat Capacity (J/(kg·°C))	Poisson’s Ratio	Thermal Conductivity (W/(m·°C))	Thermal Expansion Coefficient (°C^−1^)
Arbors, bearing caps, housings, etc.	45	210	7830	465	0.265	49.8	12.2 × 10^−6^
Motor rotor	Coppper alloy	110	8300	385	0.34	401	18.3 × 10^−6^
Motor stator	45Cr	200	7852	434	0.29	60.5	4.8 × 10^−6^
Bearing	GCr15	205	7810	553	0.3	40.11	13.3 × 10^−6^

**Table 2:** Heat generation coefficient.

Spindle Parts	Heat Generation Coefficient (W/m^3^)
Front bearing	9.824 × 10^4^
Rear bearing	1.175 × 10^4^
Motor stator	8.633 × 10^5^
Motor rotor	1.279 × 10^6^

**Table 3:** Heat transfer coefficient.

Parameter	Heat Transfer Coefficient (W/(m^2^·°C))
Front end of the rotor	68.42
Rear end of the rotor	61.28
Rotor head and air	133.2
Motor stator and coolant	400.51
Motorized spindle shell and air heat	9.7
Motor stator and rotor and air gap	138.6

**Table 4:** Experimental testing plan.

Spindle Speed (r/min)	1000	2000	3000
Time (min)	180	180	180

**Table 5:** Evaluation index model under different interference conditions.

Evaluating Indicator	R	R^2^	RMSE	MAE
No interference	0.9998	0.9996	0.2019	0.0408
Interference at measurement point 2	0.9995	0.9990	0.6538	0.1264
Interference at measurement point 3	0.9995	0.9989	0.6890	0.1314
Interference at measurement point 6	0.9995	0.9990	0.6859	0.1301
Interference at measurement point 12	0.9994	0.9989	0.7136	0.1377
All measurement points have interference	0.9975	0.9949	0.6830	0.2448

**Table 6:** Performance indicators of different algorithmic models.

Model	R	R^2^	RMSE	MAE
Ensemble learning Model	0.9975	0.9949	0.6830	0.2448
Multiple linear regression model	0.9823	0.9650	1.7963	1.4443
RBF neural network model	0.9845	0.9890	1.0062	0.7896
BP neural network model	0.9833	0.9545	2.0476	1.6087

## Data Availability

The original contributions presented in this study are included in the article. Further inquiries can be directed to the corresponding author.
